# Why stories matter in water research: A case for narrative style paper writing

**DOI:** 10.1016/j.wroa.2023.100198

**Published:** 2023-08-22

**Authors:** Treavor H. Boyer, Wolfgang Gernjak

**Affiliations:** a School of Sustainable Engineering and the Built Environment (SSEBE), Arizona State University, PO Box 873005, Tempe, AZ 85287-3005, USA; b Catalan Institute for Water Research (ICRA), Girona 17003, Spain; c Catalan Institution for Research and Advanced Studies (ICREA), Barcelona 08010, Spain

It is undeniable that our brain remembers and responds to a good story better than an isolated collection of facts (Zak, 2015). Hence, if as scientists we strive for impact, especially throughout the wider society, we should think about how to describe our research as stories. Narrative style writing is found anywhere an author is trying to tell such a story. A key feature for academic journals is that any narrative shall be supported by quantitative data or qualitative data or both.

Good non-academic examples for this can be found in sports journalism about professional baseball. In one story, the author describes how a new technical rule considering the dimensions of baseball infields does not have the same impact throughout the league (Stark, 2022). In another story, the statistical win probability of teams is ranked and discussed following the player trade deadline (Sarris, 2023b). In the third story, pitchers are ranked based on past performance metrics and projected performance (Sarris, 2023a). The point of highlighting these articles is that technical rules and advanced statistical metrics serve as the basis for these stories, but their main purpose is to support the narrative, which is what really engages the reader. As an environmental scientist aiming to write a compelling story, your challenge becomes to seamlessly weave in research implications, previous literature, and key results to convey your principal messages, *i.e.*, generate your narrative.

*Water Research X* was launched as an independent journal, separate from *Water Research*, in January 2023, and in their editorial, Editor-in-chief Eberhard Morgenroth of *Water Research* and Editor-in-chief Zhiguo Yuan of *Water Research X* describe the new journal (Yuan and Morgenroth, 2023). As a key distinctive feature of the journal as developed by the editorial team, we decided that to publish impactful research the authors of *Water Research X* shall write Leading-Edge research papers with a compelling narrative.

There are various high-level recipes and discussions available for writing narrative style papers that authors can refer to, such as Padian (2018). As a specific example, Dr. Randy Olson (Olson, 2015), advocates using what he calls the “ABT structure” derived from “AND” (present the known facts and scientific literature), “BUT” (highlight the knowledge gap or problem), and “THEREFORE” (present the solution). He distinguishes this structure from AAA (and, and, and – too many facts not linked well) and DHY (despite, however, yet – too many unresolved tensions in the writing, unclear). At the microstructure, sentences such as “Fig. 1 shows…” should be avoided. Instead, it should be “Descriptive sentence… (Fig. 1).” The latter sentence structure allows authors to focus on advancing the story rather than introducing figures and tables.

The structure of Leading-Edge manuscripts in *Water Research X* is also aimed at supporting the narrative writing style (Bade et al., 2023; Zheng et al., 2023). Directly after the Introduction comes the Results and Discussion section either combined or separate, followed by an optional Conclusions section. That way authors can tell their story in a more straightforward way. Then, the Materials and Methods section follows the Conclusions section. If it enhances the flow, and especially if required for readers to follow the narrative, authors may briefly outline key methods in the Results section to avoid the need to jump forth and back.

Leading-edge manuscripts are limited to 3000 words excluding Materials and Methods, References, figures and tables and their captions, and may contain up to 6 figures and tables. If you compare this to other journals, you will notice that the paper structure is intended to be comprehensive yet concise trying to pick the best of both worlds, *i.e.*, readability of letter-style papers and completeness of traditional full-length research papers such as published in *Water Research*.

We hope that by now, we have convinced you that adopting narrative style writing will enhance your research impact. As editors we think that by providing you with the above-described manuscript structure we gave you a good tool in hand. So now over to you as here we remain, eager to read and hopefully publish your research stories in *Water Research X*.

## Declaration of Competing Interest

The authors declare that they have no known competing financial interests or personal relationships that could have appeared to influence the work reported in this paper.

## References

[bib0001] Bade R., Rousis N., Adhikari S., Baduel C., Bijlsma L., Bizani E., Boogaerts T., Burgard D.A., Castiglioni S., Chappell A., Covaci A., Driver E.M., Sodre F.F., Fatta-Kassinos D., Galani A., Gerber C., Gracia-Lor E., Gracia-Marín E., Halden R.U., Heath E., Hernandez F., Jaunay E., Lai F.Y., Lee H.J., Laimou-Geraniou M., Oh J.E., Olafsdottir K., Phung K., Castro M.P., Psichoudaki M., Shao X., Salgueiro-Gonzalez N., Feitosa R.S., Gomes C.S., Subedi B., Löve A.S.C., Thomaidis N., Tran D., van Nuijs A., Verovšek T., Wang D., White J.M., Yargeau V., Zuccato E., Mueller J.F. (2023). Three years of wastewater surveillance for new psychoactive substances from 16 countries. Water Res. X.

[bib0002] Olson R. (2015).

[bib0003] Padian K. (2018). Narrative and “Anti-narrative” in science: how scientists tell stories, and don't. Integr. Comp. Biol..

[bib0004] Sarris, E. 2023. Starting pitcher ranks for the rest of the season. The Athletic, https://theathletic.com/4573418/4572023/4573406/4573402/sarris-starting-pitcher-ranks-4572023/. Access date: June 2, 2023.

[bib0005] Sarris, E. 2023. Which teams added the most at the MLB trade deadline – by the numbers. The Athletic, https://theathletic.com/4744013/4742023/4744008/4744003/mlb-trade-deadline-stats-numbers/. Access date: August 3, 2023.

[bib0006] Stark, J. 2022. The inside dirt: MLB addressing its 'dirty little secret' in wake of new shift rule. The Athletic, https://theathletic.com/3596022/3592022/3596009/3596015/mlb-infield-dirt-shift-rule/. Access date: September 15, 2022.

[bib0007] Yuan Z., Morgenroth E. (2023). Water Research X is becoming an independent, open access, top-quality journal in water science and technology. Water Res. X.

[bib0008] Zak, P.J. 2015. Why inspiring stories make us react: the neuroscience of narrative. Cerebrum 2015. PMC4445577 26034526

[bib0009] Zheng M., Li H., Duan H., Liu T., Wang Z., Zhao J., Hu Z., Watts S., Meng J., Liu P., Rattier M., Larsen E., Guo J., Dwyer J., Akker B.V.D., Lloyd J., Hu S., Yuan Z. (2023). One-year stable pilot-scale operation demonstrates high flexibility of mainstream anammox application. Water Res. X.

